# Public health professionals’ perceptions of mental health services in Equatorial Guinea, Central-West Africa

**DOI:** 10.11604/pamj.2016.25.236.10220

**Published:** 2016-12-13

**Authors:** Peter Robert Reuter, Shannon Marcail McGinnis, Kim Eleanor Reuter

**Affiliations:** 1 Florida Gulf Coast University, College of Health Professions and Social Work, Fort Myers, FL, 33965; 2 Public Health Management Corporation, LM 500, Lower Mezzanine, West Tower, 1500 Market Street, Philadelphia, PA 19102; 3 Temple University, Department of Biology Philadelphia, PA, 19122

**Keywords:** Equatorial Guinea, mental health, Africa

## Abstract

**Introduction:**

Mental health disorders constitute 13% of global disease burden, the impacts of which are disproportionality felt in sub-Saharan Africa. Equatorial Guinea, located in Central-West Africa, has the highest per-capita investment in healthcare on the African continent, but only two studies have discussed mental health issues in the country and none of have examined the perspective of professionals working in the field. The purpose of this study was to gain a preliminary understanding of Equatoguinean health care professionals' perspectives on the mental health care system.

**Methods:**

Nine adult participants (directors or program managers) were interviewed in July 2013 in Malabo, Equatorial Guinea from government agencies, aid organizations, hospitals, and pharmacies. Interviews were designed to collect broad information about the mental healthcare system in Equatorial Guinea including the professionals' perspectives and access to resources. This research was reviewed and approved by an ethical oversight committee.

**Results:**

All individuals interviewed indicated that the mental health system does not currently meet the needs of the community. Professionals cited infrastructural capacity, stigmatization, and a lack of other resources (training programs, knowledgeable staff, medications, data) as key factors that limit the effectiveness of mental healthcare.

**Conclusion:**

This study provides a preliminary understanding of the existing mental health care needs in the country, highlighting opportunities for enhanced healthcare services.

## Introduction

Mental health disorders constitute 13% of the global disease burden [1] and will result in a lost global economic output of US$16.1 trillion by 2030 [2]. Impaired mental health is associated with poverty, marginalization, social disadvantage, and substance and alcohol abuse [3–5]. However, global spending on mental health is less than US$2 per person, per year and many governments spend less than 1% of the total health expenditure on mental health, leaving a funding and treatment gap [6]; in low-income and high-income countries, 76-85% and 35-50% people, respectively, receive no treatment for their disorder [5].

Funding for healthcare in Africa is lower than in other parts of the world; 4% of the Gross Domestic Product (GDP) is spent on healthcare, compared to 7% in Europe and the Americas [3]. In addition, fewer African World Health Organization (WHO) member countries have adopted mental health policies (42%) than countries in all other WHO regions including the Americas (56%), Eastern Mediterranean (68%), Europe (73%), and South-East Asia (70%), and Western Pacific (58%) [6]. Data are scarce on the prevalence of mental illness throughout much of Africa [7], but country-specific studies have found high burdens of mental disorders in several countries (12% in Nigeria, [8]; 13% in the Gambia, [9]; and 30.3% in South Africa, inclusive of substance abuse, [10]).

Equatorial Guinea (Central-West Africa) is one of the smallest countries in Africa [11] with one of the fastest growing economies [12]. In 2010, Equatorial Guinea invested $612 per capita in healthcare, the highest per-capita investment on the African continent followed by the Seychelles ($338) and South Africa ($294) [13]. Equatorial Guinea has made recent progress in its healthcare provision by constructing and renovating hospitals and health care centers and instituting national socioeconomic development plans [14]. In terms of mental healthcare, there were plans to build two in-country neuropsychiatric hospitals in 2009 and in 2010 the country implemented its first mental health policy [4]. However, in spite of the country's recent efforts, Equatorial Guinea's highly centralized health care system is largely based in the major cities where only 39% of the population resides [4], leading to inequities in health care between urban and rural communities [4, 15]. Since the adoption of Equatorial Guinea's mental health policy, little has been done to assess the state of mental health care within Equatorial Guinea. Only two published peer-reviewed articles discuss mental health in Equatorial Guinea [4, 16] and no studies have assessed mental health in Equatorial Guinea from the perspective of professionals working in the field. Therefore, using social survey methods, we sought to gain a preliminary understanding of Equatoguinean professionals' perspectives on the mental health care system.

## Methods

**Recruitment:** A total of nine adult participants (who as a result of their professional responsibilities were expected to be aware of regional and national mental health services) were recruited from a broad range of health organizations (hospitals, pharmacies, clinics, international aid organizations, government entities). In order to gain a comprehensive understanding of the national mental health system, we secured interviews with directors or program managers in government agencies and large multi-national aid organizations, as well as with directors of hospitals (public and private) and pharmacies (public and private). Recruitment occurred either face-to-face at their workplace or over the phone. All participants were based in the capital city, Malabo, although some managed projects with national impact. At the time of data collection, there were no more than 50 individuals in the country with the same standing in the healthcare field as the 9 individuals interviewed here. All respondents had been in their present position for an average of 9 ± 7 years (mean ± st. dev). Given concerns regarding respondents' privacy and anonymity, no further information about their professional responsibilities can be provided.

**Survey methods:** We administered semi-structured interviews [17] in July 2013 in Malabo. Interviews lasted 25 ± 12 minutes (range: 10-49 min.) and were conducted in private, at a time and place determined by the respondents. All interviews were conducted in Spanish by one Spanish-speaking interviewer who was not known to the respondent and was not local to the area. No incentives were provided to respondents. Respondents could choose not to answer any question.

**Survey instrument:** Interviews were designed to collect broad information about the mental healthcare system in Equatorial Guinea including the professionals' perspectives and access to resources. Questions included the following: 1) What are your views on the need for mental health treatment?; 2) At your work, is there access to medications (such as anti-depressants, anti-epileptics, etc.), information and resources about mental health or psychology, and/or psychology or mental health professionals, including support groups for patients?; 3) Is mental health a part of the academic curriculum in schools?; 4) What postgraduate education programs exist for those who want to study psychology or mental health?; and 5) Was mental health training part of your education?

**Ethical considerations:** Though identifying information was collected, respondents remain anonymous in this paper. This research was reviewed and approved by an ethical oversight committee (Institutional Review Board, Florida Gulf Coast University, Protocol ID Number: 2012-26) and was conducted with permission from the University of Equatorial Guinea Medical School. All interviews were conducted by a researcher trained in ethical data collection. Written consent was secured.

**Statistical analysis:** Interviews were recorded, transcribed by the interviewer, and translated into English by a volunteer, native Spanish speaker. Given the small sample size, data were not coded or analyzed through qualitative research software. Because all questions were meant to gain a broad understanding of existing needs, results are not presented for each question but instead, key themes were identified by the research team and summarized.

## Results

**Perceptions on mental health needs in Equatorial Guinea:** All respondents indicated a need for improved mental health services in Equatorial Guinea, describing the need as “urgent”, “necessary”, and “important”. Respondents specified that “there are people that do not receive treatment” (including within the urban areas of Malabo and Bata) and “there are many and various mental health problems”.

**Additional or improved infrastructure:** Two-thirds of respondents described a lack of infrastructure for treating mentally ill patients, but differed in their knowledge of existing infrastructure. Three respondents referenced existing centers designed to treat the mentally ill, while three others thought a center is needed and there are plans to build one. Among those that referenced existing centers, two described the centers as ineffective (the centers are “the only program in the Ministry that does not work”) and felt that “more support” is needed because the centers were “not appropriate” for the needs of mentally ill patients.

Specifically one respondent said:

“They need to find a safe place when we do treatments in case the patients are angry or have a reaction to the meds. People can get violent and jump out the window… We need more security to help”.

No respondents listed outpatient or informational resources available specifically for mental health programs though two described existing in-country resources for AIDS, alcoholism, and smoking cessation. Despite this, one respondent was optimistic about the current progress with a feeling that the “infrastructure is starting to work”.

**Reducing stigma:** One-third of respondents referenced a need to address stigmatization and a lack of public understanding around mental health disorders. One respondent described the general population as “ignorant” towards mental health disorders. Almost half (n=4) explained that mental health is viewed as “taboo” and mental health disorders are thought of as a “curse”, “demonic state”, or “witchcraft”. Another respondent discussed how this lack of understanding could influence attitudes towards the mentally ill explaining, “when you don't understand something, usually you feel scared and you want it to go away”. Respondents felt these views cause society to “reject” those with mental health disorders and influence the way society perceives treatment options, as one respondent stated:

“The perception of a mental hospital is that it is a place similar to a jail, a place where people are abandoned by everyone. We have many schizophrenics on the streets and the idea is to put them in a place and lock them up, throw away the keys”.

Although stigmatization may indicate a need for improved public education, one respondent thought that some information was already being shared with the public about mental health and described advertisements in which a person is treated for a mental health disorder and then is shown “having a normal life”.

**Increased data and information:** There were some incongruencies in respondents' understanding of the availability of data on mental health. One respondent indicated that Equatorial Guinea had previously received assistance from the Spanish government to collect data on mental illness and conduct “an analysis of the psychology of diseases, the situation, policy, and illness as any other disease”. Two other respondents referenced a lack of data available on the mental health burden in, and mental health service needs of, Equatoguinean communities (with one stating the need to “ask for cooperation with the NGOs” to conduct an initial survey on mental illness). Two further respondents explicitly noted the importance of collecting data to identify individuals with mental health disorders. In addition to local data on mental health disorders, most respondents (n=7) indicated they had no, “very little”, or “not enough” access to information and resources about general mental health in their workplace and that this problem was nation-wide. Respondents felt this lack of available information negatively influenced the quality of care in Equatorial Guinea by limiting healthcare professionals' knowledge about the existence of, or side effects from, certain medications and their ability to recognize how symptoms manifest in different individuals.

**Additional government assistance:** When discussing mental health service needs, only one respondent spoke specifically about the country's mental health policy, stating:

“I think that mental health treatment in our country is not being handled or implemented appropriately. The mental health policy in this country needs work. We need to increase awareness and make a viable action plan. A lot of plans are made and then they end up in a drawer and money is spent but the problem is a difficult one”.

Another respondent alluded to a need for an investment in additional government resources to address the “structure” of the Ministry of Health, concluding that “the country is rich, but people are not” and that wealth disparity (and associated high rates of poverty) are “the problem”.

**Access to resources:** Respondents were asked about their access to resources/information in their workplace as well as how in-country academic curricula or educational programs addressed mental health. Some participants were unable to provide comprehensive answers to certain questions because they either did not work for organizations that provided direct patient care or they had been educated outside the country.

**Access to medications:** Five respondents worked in facilities that have access to medications for mental health disorders; two of these worked for organizations that employ a mental health specialist or psychiatrist. In addition, two respondents worked for organizations that had lost the ability to provide medications, having received assistance from NGOs or outside organizations in the past (and expressing a wish for future assistance to make relevant medications available). One respondent explained that assistance from outside organizations was not sustainable because they “help for a specific time but then they have to leave”. Another respondent explained how political and economic barriers limit access to relevant medications:

“ … if it is a center that is going to import the drug, they will ask if it is licensed, if it really is a center. Then, it is difficult. It is difficult because entry standards that the exporting country asks for are difficult to fulfill. If the medical center cannot fulfill these rigorous standards, then they cannot offer it to their patients. It is more a regulatory problem more than anything from the exporting country.”

This respondent added that these issues lead to a shortage of available medications for the mentally ill because it is “not financially beneficial for pharmacies to carry these types of drugs”. This conclusion is supported by another respondent, a pharmacist, who described how some prescriptions are “easy to stock but others are not”. Another respondent (also a pharmacist) expressed reluctance in prescribing psychotropic medications because patients often take incorrect doses.

**Access to mental health specialists and professionals:** Five respondents referenced a lack of available specialists or doctors to treat mental health disorders, but gave varying estimates of the number of available specialists in-country. Some respondents said there were “no specialists” while others thought that “psychologists exist” in cities such as Bata, but they are “deficient at the national level” (also stating “we need more personnel”). One respondent thought there is “only one psychologist for the whole island” referring to Bioko Island where the capital city (Malabo) is located. Two thought there were two psychologists or mental health professionals in all of Equatorial Guinea, another thought there were two in Malabo. Four respondents worked directly with mental health specialists and one made direct reference to a psychiatrist in a general hospital setting, noting “when we have a case, we send it there”. In response to this shortage, one respondent thought the government was “creating specialists” as a part of their plan to build treatment centers, whereas another thought “there are no psychologists in training now”.

**Access to education and training:** Four respondents indicated that formal educational opportunities on mental health were not available in Equatorial Guinea. One respondent said, “we do have a school of nursing and a school of medicine. But there are no specialties” available in-country. Additional training needs were further articulated by one respondent related to prescribing medications:

“There needs to be training in order to become a prescriber (of mental health medications). You know that in Europe, a nurse has no right to give these types of medications. Here they do. It means that they have not had training or preparations. It means there is a problem of training. People do everything without training and it is dangerous”.

In order to increase the amount of mental health specialists and to create better treatment options, respondents felt that “the University needs to prepare more psychologists”.

## Discussion

Using semi-structured interviews with health professionals in Equatorial Guinea, we present findings on their perceptions of mental health treatment in the country. Our findings highlight several key perceived needs within Equatorial Guinea and may advise future mental health policy and research in Equatorial Guinea.

**Perceptions on mental health needs in Equatorial Guinea:** Respondents were unanimous in their view that the current medical system is not meeting the mental health needs of the community. Specific needs identified by respondents included additional or improved infrastructure, reducing stigmatization, improved data and information, and additional governmental assistance.

**Additional or improved infrastructure:** Physical infrastructure designed to treat the mentally ill is necessary to meet the needs of this group [18]. Respondents discussed a lack of physical infrastructure in Equatorial Guinea which has also been identified across other African countries (e.g. Nigeria, [19]; Liberia, Sierra Leone, the Gambia, [20]). In addition to limiting the number of available services, a lack of physical infrastructure increases the travel distance required to receive appropriate care, which is a barrier for some patients or caregivers who are unable to invest the resources into long-distance travel [19]. When discussing existing physical infrastructure in Equatorial Guinea, respondents had inconsistent views on whether or not mental health-specific infrastructure exists. These inconsistencies may point towards a lack of information sharing within the healthcare system and a lack of awareness among professionals.

**Reducing stigma:** The stigmatization of mental health disorders in Equatorial Guinea is worrying but consistent with those expressed in other areas of the world (Africa, [19–21]; Europe, [22]; United States of America, reviewed by [23]). Stigmatization of mental illness can decrease the use of resources [24] and help-seeking behaviors [19], and increase the risk of violent victimization [25]. Strategies to reduce stigmatization may involve public education and involving the community in mental health care [7] which can be done through advocacy efforts and better media coverage of mental health issues [26].

**Improved data and information:** Comprehensive data on the mental health burden are necessary for implementing policy [7, 26]. In addition, the perception that indicators of mental health are weak, has been cited as a reason for a lack of attention and available funding for mental health globally [27]. We were only able to find two other published articles on mental health in Equatorial Guinea. The first was a review article citing undated information [16] and the other presented data from 2008 and 2009 [4]. The reasons for this lack of data are complex but may be linked to the country´s closed borders until the late-1970´s [28] and the lack of reliable baseline data [29]. It is also noteworthy that our respondents, despite their elevated positions within the healthcare community, did not have access to information on general mental health issues at their workplace. Factors that may contribute to a lack of mental health data include a large proportion of patients seeking care from traditional medicine practitioners and a lack of health workers trained in data collection [26]. More available data on mental health may help increase awareness and understanding among health workers and encourage advocacy and policy action among decision makers [26].

**Additional governmental assistance:** Equatorial Guinea implemented its first mental health policy in 2010 [4]. As 58% of African countries do not have a mental health policy, and 33% do not have a mental health plan [6], this was a significant step towards addressing the country's mental health needs. However, for this policy to be successful, there must be appropriate dissemination and operationalization to prevent the weak implementation that has been seen in other African countries including Ghana and Zambia [4, 30]. Several respondents addressed the need for better organization and investment in resources by the government to improve mental health care in Equatorial Guinea, but only one referenced the policy directly. Because so few respondents spoke specifically about this policy, it is unclear whether its implementation has been successful. Further, the inequalities and inequities in care mentioned by respondents are similar to those articulated in other African communities [3, 31] and around the world (e.g. in the United States, those classified as low income have 1.5 times the odds of having an unmet need for mental health services than those with higher incomes, [32]). The relationship between poverty and unmet need for mental health services could be in part due to: 1) a delayed disease prognosis among those who cannot afford care, which may exacerbate symptoms; 2) an increased risk of mental illness among individuals in poverty; and 3) fewer available resources in impoverished societies that can increase staff shortages, decrease physical infrastructure, and lead to poor health training for providers [3]. Each of these points were mentioned by at least one respondent. Additional government assistance through advocacy, integration, and legislation, could improve mental health care for individuals in more rural and poorer sectors of the community [18].

**Access to medications:** A lack of medications to treat mental health disorders is an obstacle facing mental health systems in Equatorial Guinea [4] and elsewhere [7, 20]. Access to these medications is limited in countries across Africa due to inconsistent supply, high prices [20], and poor purchasing power of these countries [7]. In Equatorial Guinea, respondents attributed the lack of available drugs, in part, to the strict regulatory limitations imposed on clinics. However, these regulations have not suppressed the availability of drugs used to treat other illnesses including Malaria or childhood vaccinations, for which there is a higher and constant demand. Therefore, the lack of drugs used to treat mental illness may be because of a lack of demand, which may be due to: 1) a lack of community recognition of mental health as a treatable, medical issue; 2) low levels of outreach to communities and few out-patient facilities that prescribe these medications; and 3) a lack of healthcare workers who are trained in prescribing these medications [5].

**Access to trained mental health care specialists and professionals:** The lack of mental health specialists has been identified previously in Equatorial Guinea [4] and across Africa [3, 7]. In 2014, Sierra Leone had only one retired psychiatrist for six million people, and in the same year Nigeria had 160 psychiatrists for 160 million people [20]. Furthermore, across nine other African countries, “the provision of mental health care” was one of the least common carried out job functions reported by nurses and midwives [33]. According to the WHO, almost half the world´s population lives in countries that have on average, one psychiatrist to serve 200,000 or more people [5]. Respondents noted that in order to specialize in mental health, one would have to be trained outside the country, which may cause these professionals to seek work elsewhere to avoid relocation. One method to increase the number of trained professionals is to provide basic training programs for non-specialist healthcare workers. Psychological treatment by non-specialist healthcare providers in developing countries has been found to be effective in treating some types of depression [34] and could provide a rapid, but effective, increase in the capacity of the Equatoguinean mental health system. Other methods that have been identified to increase the number of trained professionals in Africa includes improving working conditions for these professionals and providing incentives to become trained [7].

## Conclusion

If the estimates of mental health prevalence in Equatorial Guinea [4] and in Africa as a whole [21] are true, thousands of individuals are currently suffering from a mental illness with limited access to treatment options. Among our respondent pool, there was an acceptance that the current mental health system in Equatorial Guinea does not adequately address the needs of communities. Although infrastructural capacity for mental health services may have recently increased in Equatorial Guinea, the lack of other resources (including training programs, knowledgeable staff, medications, and community support) may limit the effectiveness of these facilities and information-sharing among health professionals.

While we acknowledge the limitations of our study, the elevated professional appointments held by our respondents allowed for a broad, preliminary understanding of the existing mental health care needs in the country. Additional research is needed to fully assess the status and needs of the mental health community in Equatorial Guinea. It is unclear what percentage of individuals in Equatorial Guinea currently suffer from mental illness, and whether segments of the population are at a higher risk than others. In addition, interviews with traditional healthcare workers may provide insight into other ways of seeking care for mental health disorders in the country.

### What is known about this topic

- Mental health disorders are a global public health burden, however most countries invest very little in their mental health system and few countries have a mental health policy;
- Equatorial Guinea implemented its mental health policy in 2010 and has the highest per-capita investment in healthcare compared to other African Counties.

### What this study adds

- Since the adoption of its mental health policy, this study found that health care professionals still believe there are unmet needs in the country;
- Specific factors that may contribute to unmet mental healthcare needs in Equatorial Guinea may include infrastructural capacity, stigmatization, a lack of other resources such as training programs, knowledgeable staff, medications, community support, and poor information-sharing among health professionals.

## References

[1] Canavan ME, Sipsma H, Adhvaryu A, Ofori-Atta A, Jack H, Udry C, Osei-Akoto I, Bradley EH (2013). Psychological distress in Ghana: associations with employment and lost productivity. International Journal of Mental Health Systems..

[2] World Economic Forum, Harvard School of Public Health (2011). The Global Economic Burden of Noncommunicable Diseases..

[3] De-Graft-Aikins A, Marks DF (2007). Health, Disease and Healthcare in Africa. Journal of Health Psychology..

[4] Morón-Nozaleda MG, de Tojeiro JG, Cobos-Muñoz D, Fernández-Liria A (2011). Integrating mental health into primary care in Africa: the case of Equatorial Guinea. Intervention..

[5] World Health Organization (2013). Mental Health Action Plan 2013-2020..

[6] World Health Organization (2011). Mental health atlas 2011..

[7] Gureje O, Alem A (2000). Mental health policy development in Africa. Bulletin of the World Health Organization..

[8] Gureje O, Lasebikan VO, Kola L, Makanjuola VA (2006). Lifetime and 12-month prevalence of mental disorders in the Nigerian Survey of Mental Health and Well-Being. British Journal of Psychiatry..

[9] (2012). Mental Health Leadership and Advocacy Programme (mhLAP): Gambia..

[10] Herman AA, Stein DJ, Seedat S, Heeringa SG, Moomal H, Williams DR (2009). The South African Stress and Health (SASH) study: 12-month and lifetime prevalence of common mental disorders. South African Medical Journal..

[11] Central Intelligence Agency The World Factbook: Equatorial Guinea..

[12] The World Bank Equatorial Guinea..

[13] Africa Health, Human & Social Development Information Service and Parliamentary Support Network (2013). Africa Health Financing Scorecard- Featuring Year 2000-2010 Indicative Progress Summary..

[14] World Health Organization Country Cooperation Strategy at a glance. Equatorial Guinea..

[15] Romay-Barja M, Jarrin I, Ncogo P, Nseng G, Sagrado MJ, Santana-Morales MA, Aparcio P, Valladares B, Riloha M, Benito A (2015). Rural-Urban Differences in Household Treatment-Seeking Behaviour for Suspected Malaria in Children at Bata District, Equatorial Guinea. PLoS One..

[16] Jacob KS, Sharan P, Mirza I, Garrido-Cumbrera M, Seedat S, Mari JJ, Sreenivas V, Saxena S (2007). Mental health systems in countries: where are we now?. The Lancet..

[17] Rietbergen-McCracken J, Narayan-Parker D (1996). Participation and Social Assessment: Tools and Techniques Volume 1..

[18] Szabo CP (2013). Editorial: Improving Mental Health Systems in Africa. African Journal of Psychiatry..

[19] Jack-Ide IO, Uys L (2013). Barriers to mental health services utilization in the Niger Delta region of Nigeria: service users' perspectives. The Pan African Medical Journal..

[20] Esan O, Abdumalik J, Eaton J, Kola L, Fadahunsi W, Gureje O (2014). Mental Health Care in Anglophone West Africa. Psychiatric Services..

[21] Travers KU, Pokora TD, Cadarette SM, Mould JF (2013). Major depressive disorder in Africa and the Middle East: a systematic literature review. Expert Review of Pharmacoeconomics & Outcomes Research..

[22] Crisp AH, Gelder MG, Rix S, Meltzer HI, Rowlands OJ (2000). Stigmatisation of people with mental illnesses. The British Journal of Psychiatry..

[23] Parcesepe AM, Cabassa LJ (2013). Public stigma of mental illness in the United States: a systematic literature review. Administration and Policy in Mental Health and Mental Health Services Research..

[24] Saxena S, Thornicroft G, Knapp M, Whiteford H (2001). Resources for mental health: scarcity, inequity, and inefficiency. The Lancet..

[25] Tsigebrhan R, Shibre T, Medhin G, Fekadu A, Hanlon C (2014). Violence and violent victimization in people with severe mental illness in a rural low-income country setting: A comparative cross-sectional community study. Schizophrenia Research..

[26] Bird P, Omar M, Doku V, Lund C, Nsereko JR, Mwanza J (2011). Increasing the priority of mental health in Africa: findings from qualitative research in Ghana, South African, Uganda, and Zambia. Health Policy and Planning..

[27] Saraceno B, van Ommeren M, Batniji R, Cohen A, Gureje O, Mahoney J, Sridhard D, Underhill C (2007). Barriers to improvement of mental health services in low-income and middle-income countries. The Lancet..

[28] Brown RC (1980). Collapse of a medical system: the case of Equatorial Guinea. Annals of Internal Medicine..

[29] World Health Organization (2009). World Health Statistics 2009..

[30] Omar MA, Green AT, Bird PK, Mirzoev T, Flisher AJ, Kigozi F, Lund C, Mwanza J, Ofori-Atta AL (2013). Mental health policy process: A comparative study of Ghana, South Africa, Uganda and Zambia. International Journal of Mental Health Systems..

[31] Jenkins R, Othieno C, Okeyo S, Aruwa J, Kingora J, Jenkins B (2013). Health system challenges to integration of mental health delivery in primary care in Kenya-perspectives of primary care health workers. BMC Health Services Research..

[32] Roll JM, Kennedy J, Tran M, Howell D (2013). Disparities in unmet need for mental health services in the United States, 1997-2010. Psychiatric Services..

[33] Uys L, Chipps J, Kohi T, Makoka D, Libetwa M (2012). Role analysis of the nurse/midwives in the health services in Sub-Saharan Africa. Journal of Advanced Nursing..

[34] Chowdhary N, Sikander S, Atif N, Singh N, Ahmad I, Fuhr DC, Rahman A, Patel V (2014). The content and delivery of psychological interventions for perinatal depression by non-specialist health workers in low and middle income countries: A systematic review. Best Practice & Research Clinical Obstetrics and Gynaecology..

